# Integrative Assessment of Evidence for Anagenetic Speciation in Ulleungdo Endemic Plants: Plastid, Nuclear, and Morphological Perspectives

**DOI:** 10.3390/genes17091109

**Published:** 2026-09-12

**Authors:** Sajid Ali, Adnan Amin

**Affiliations:** 1 Department of Horticulture and Life Science, Yeungnam University, Gyeongsan 38541, Republic of Korea; 2 Department of Life Sciences, Yeungnam University, Gyeongsan 38541, Republic of Korea

**Keywords:** chloroplast genome, anagenesis, cytonuclear discordance, island endemism, species delimitation

## Abstract

Ulleungdo Island harbors several endemic plant lineages commonly interpreted as products of anagenetic speciation following long-distance colonization. Yet plastome phylogenies represent a single organellar genealogy and may conflict with nuclear genomic structure, morphology, or taxonomic boundaries. This critical mini-review evaluates whether plastid-based hypotheses for the origin and diversification of Ulleungdo endemics are supported by independent molecular and phenotypic evidence. We synthesize findings from comparative plastomics, chloroplast haplotype and network analyses, nuclear microsatellites, nrDNA sequencing, genome-wide multiplexed inter-simple sequence repeat genotyping by sequencing (MIG-seq) single-nucleotide polymorphisms (SNP), cytogenetics, morphology, and multicompartment phylogenomics. The evidence reveals heterogeneous evolutionary outcomes. *Prunus takesimensis* and *Phedimus takesimensis* are broadly consistent with probable single-origin scenarios, although confidence is constrained by progenitor sampling and marker resolution. *Rubus takesimensis* exhibits chloroplast non-monophyly and elevated haplotype diversity consistent with multiple maternal origins, whereas *Acer takesimense* shows signatures of historical drift and loss of rare nuclear alleles. Morphological, chromosomal, plastid, and nuclear–ribosomal evidence strongly support *Allium ulleungense*, while the delimitation of *Viola ulleungdoensis* remains unresolved. Plastid–nuclear incongruence in *Hepatica maxima* and *Fagus multinervis* further indicates that introgression, hybridization, incomplete lineage sorting, and restricted taxon sampling can complicate evolutionary inference. This comparative framework distinguishes species histories from provisional interpretations based on limited accessions or uniparentally inherited markers. The novelty of this review lies in its cross-taxon assessment of plastome–nuclear–morphological congruence rather than a descriptive plastome inventory. Ulleungdo lineages interpreted as products of anagenetic speciation do not exhibit a uniform genomic signature and that robust taxonomic, evolutionary, and conservation inference requires population-level integration of independently inherited genomic compartments with standardized morphological and reproductive data.

## 1. Introduction

Oceanic islands provide tractable systems for examining how long-distance colonization, isolation, and divergence generate endemic lineages [1]. Ulleungdo was selected because it is a volcanic oceanic island with numerous endemic plant lineages that have historically been interpreted as products of anagenesis, enabling cross-taxon comparisons within a shared insular setting [2] (Lee et al., 2017). Its environmental history is also documented: Ulleungdo belongs to Korea’s maritime warm floristic zone [3] (Jung & Cho, 2020), and tephrostratigraphic studies have reconstructed its Late Quaternary volcanic history [4] (McLean et al., 2020). Comparable anagenetic patterns occur in other oceanic archipelagos, although their outcomes depend on local ecological and geological context [5,6]. This model does not require a uniform population-genetic outcome across taxa. Studies of Ulleungdo endemics have instead recovered contrasting patterns of diversity, structure, and ancestry, indicating that the genetic consequences of presumed anagenesis must be evaluated empirically for each lineage [7,8].

Morphological differentiation, plastid lineage differentiation, nuclear genomic differentiation, and species delimitation are not interchangeable [9,10]. Plastid markers describe the history of one genomic compartment and, where the mode of plastid inheritance is established, can inform maternal-lineage and seed-mediated colonization hypotheses [11,12]. Duan et al. directly used plastomes to identify maternal origins, while Setsuko et al. combined chloroplast and nuclear evidence to examine dispersal and migration among oceanic islands [11]. Nuclear markers provide independently inherited information for evaluating population structure, admixture, and lineage differentiation [13]. Morphological and cytogenetic data provide additional evidence for phenotypic diagnosability and taxonomic differentiation [14]. Congruence among these evidence classes strengthens an evolutionary interpretation [15], whereas discordance requires alternative explanations or additional sampling [16]. Xiao et al. directly demonstrated plastid–nuclear discordance associated with incomplete lineage sorting and probable interspecific hybridization in Clematis [16]. Present-day genetic diversity also cannot be treated as a direct measurement of the initial founder event because subsequent mutation, recombination, drift, migration, and demographic change can modify colonization signatures [17].

Empirical results from Ulleungdo demonstrate this heterogeneity. Nine nuclear microsatellite loci distinguished *Acer takesimense* from *A. pseudosieboldianum* and revealed slightly lower genetic diversity, less than half the number of private and rare alleles, and weak within-island geographical structure, a pattern interpreted as evidence of historical drift [18]. *Phedimus takesimensis* was monophyletic in the sampled nuclear ribosomal internal transcribed spacer and chloroplast regions and showed no apparent reduction in the assessed genetic-diversity measures relative to the examined continental relatives; however, the authors cautioned that uneven progenitor sampling constrained this comparison [8]. In contrast, broad chloroplast sampling of *Rubus takesimensis* recovered non-monophyly, 48 haplotypes restricted to the island endemic, and contributions from geographically differentiated continental populations, supporting multiple maternal origins rather than a simple single-colonization history [19]. Genome-wide multiplexed inter-simple sequence repeat genotyping by sequencing (MIG-seq) single-nucleotide polymorphisms, together with chloroplast DNA haplotypes, supported monophyly, differentiation from *P. sargentii*, and a probable single colonization of *Prunus takesimensis*, although its precise geographical source remained unresolved [7].

The limitations of single-compartment inference are especially evident in *Hepatica maxima*. Phylogenomic analyses of 76 plastid, 37 mitochondrial, and 413 nuclear genes recovered cytonuclear and organellar discordance, while species-network analyses supported ancient and more recent introgression within the evolutionary history of the lineage [20]. These findings demonstrate that a plastid topology may represent only one component of species history when reticulation or incomplete lineage sorting contributes to phylogenetic discordance [21]. The principal research gap is therefore not simply a shortage of molecular data, but the absence of a cross-taxon synthesis that evaluates plastid-based hypotheses against independently inherited nuclear evidence, morphology, cytogenetics, and population-level sampling using explicit and comparable inferential criteria.

This critical mini-review addresses that gap by asking whether presumed anagenetic Ulleungdo endemics exhibit consistent genetic consequences, whether plastid relationships are congruent with nuclear and morphological evidence, which taxonomic and evolutionary interpretations are strongly supported or remain provisional, and what evidence is required before genomic findings can inform conservation decisions. The cross-compartment comparisons required at least two distinct classes of evidence, whereas population-level single-compartment studies were retained only when they directly tested a defined evolutionary hypothesis. Single-accession plastome reports are treated as phylogenetic resources rather than as population-level tests of colonization history, genetic diversity, or species boundaries.

## 2. Literature Search and Evidence Classification

This critical review searched PubMed, Scopus, Web of Science, and Google Scholar from the inception of each database through May 2026. Searches combined “Ulleungdo,” “anagenetic speciation,” and focal taxon names with terms related to plastid and nuclear genomics, phylogeny, population genetics, morphology, cytogenetics, hybridization, and introgression. Primary studies on Ulleungdo endemics or putative continental relatives were screened by title/abstract and full text; duplicate, irrelevant, and insufficiently informative records were excluded. For each eligible study, taxon identity, sampling design, evidence type, principal inference, and major limitations were extracted.

Independent evidence classes comprised plastid, mitochondrial, nuclear, morphological, and cytogenetic data, with multiple loci from the same genomic compartment treated as one class. Comparative or population-level datasets directly testing evolutionary hypotheses were distinguished from genomic resources, such as single-accession plastomes or reference genomes without comparative validation. Single-class evidence was used for question-specific inference, whereas cross-compartment assessment required at least two independent evidence classes. Anagenetic speciation was treated as a composite hypothesis, with taxonomic distinctness, colonization history, progenitor identity, within-island divergence, hybridization/introgression, and demographic change assessed separately. Evidence was classified as congruent when independent datasets supported the same inference, partially congruent when they agreed on the principal inference but differed in secondary interpretation, discordant when independent datasets supported incompatible conclusions for the same question, and unresolved when evidence was missing or insufficient. These criteria were applied consistently in the taxon-level synthesis. Evidence strength was graded independently as high, moderate, or low according to marker resolution, population-level sample representation, and geographic/source-taxon coverage.

## 3. Evidence Types and Limits of Inference

### 3.1. Single-Accession Plastomes Versus Population-Level Chloroplast Data

A complete plastome can characterize genome organization, gene content, structural variation, and plastid-based phylogenetic placement [22]. A plastome from a single individual, however, represents a single sampled chloroplast lineage and cannot estimate within-species haplotype diversity, population structure, or demographic history. These questions require population-level sampling [23]. Plastid loci were treated as a single linked evidence class because no focal Ulleungdo study reviewed here has demonstrated interplastomic recombination. Although interplastomic recombination can generate region-specific plastid histories in exceptional lineages [24,25], recent analyses show that such events should be inferred only when intraplastomic phylogenetic conflict is explicitly demonstrated and alternative sources of gene-tree conflict are evaluated [26]. Single-accession plastomes should therefore be treated as genomic and phylogenetic resources, not as population-level tests of colonization or diversification.

### 3.2. Population-Level Chloroplast Evidence

Population-level chloroplast data can quantify haplotype diversity, test chloroplast monophyly, reveal geographic haplotype structure, and identify distinct chloroplast lineages among sampled populations [27]. Such data can constrain hypotheses of seed-mediated colonization when plastid inheritance is maternal. Maternal transmission must, however, be established or biologically justified for the focal lineage [28]. Experimental studies detected low-frequency paternal plastid transmission in *Arabidopsis thaliana* and biparental plastid inheritance in *Pelargonium* hybrids [29,30]. Chloroplast haplotypes should therefore be interpreted as maternal-lineage markers only when the assumption of maternal inheritance is supported.

### 3.3. Nuclear Markers and Genome-Wide Evidence

Nuclear marker systems differ substantially in genomic coverage and resolution. Microsatellites estimate allelic diversity and population structure across a limited number of loci [31]. Nuclear ribosomal internal transcribed spacer (nrDNA ITS) sequences can inform phylogenetic relationships, but their multicopy nature and concerted evolution may obscure parental variants following hybridization, as demonstrated experimentally and in natural hybrid systems [32]. Low-copy nuclear genes provide independent loci but require assessment of orthology and paralogy [33]. Multiplexed inter-simple sequence repeat genotyping by sequencing (MIG-seq) is a polymerase chain reaction-based reduced-representation method that generates genome-wide single-nucleotide polymorphisms without prior genomic information [34]. Its resolution remains dependent on locus recovery, filtering, missing data, and sampling design. Transcriptome samples capture nuclear gene expression, whereas whole-genome datasets provide a broader genomic representation.

### 3.4. Morphological and Cytogenetic Evidence

Morphological evidence is strongest when diagnostic characters are consistently recovered across adequately sampled populations [35]. Environmentally responsive traits require additional testing before they are treated as stable taxonomic differences. Chromosome number and ploidy provide useful cytological characterization but do not necessarily constitute species-level cytogenetic differentiation when closely related taxa share the same chromosome complement. Cytogenetic evidence is more discriminatory when comparative differences involve chromosome number, ploidy, karyotype architecture, chromosomal rearrangements, or chromosome-specific markers [36,37]. For example, *Allium ulleungense* was distinguished from close relatives using morphological characters, diploid chromosome number, and both nuclear and chloroplast markers [38]. Cytogenetic differentiation alone, however, does not identify a progenitor lineage or establish a particular mode of speciation.

### 3.5. Interpretive Framework

**Figure 1:**
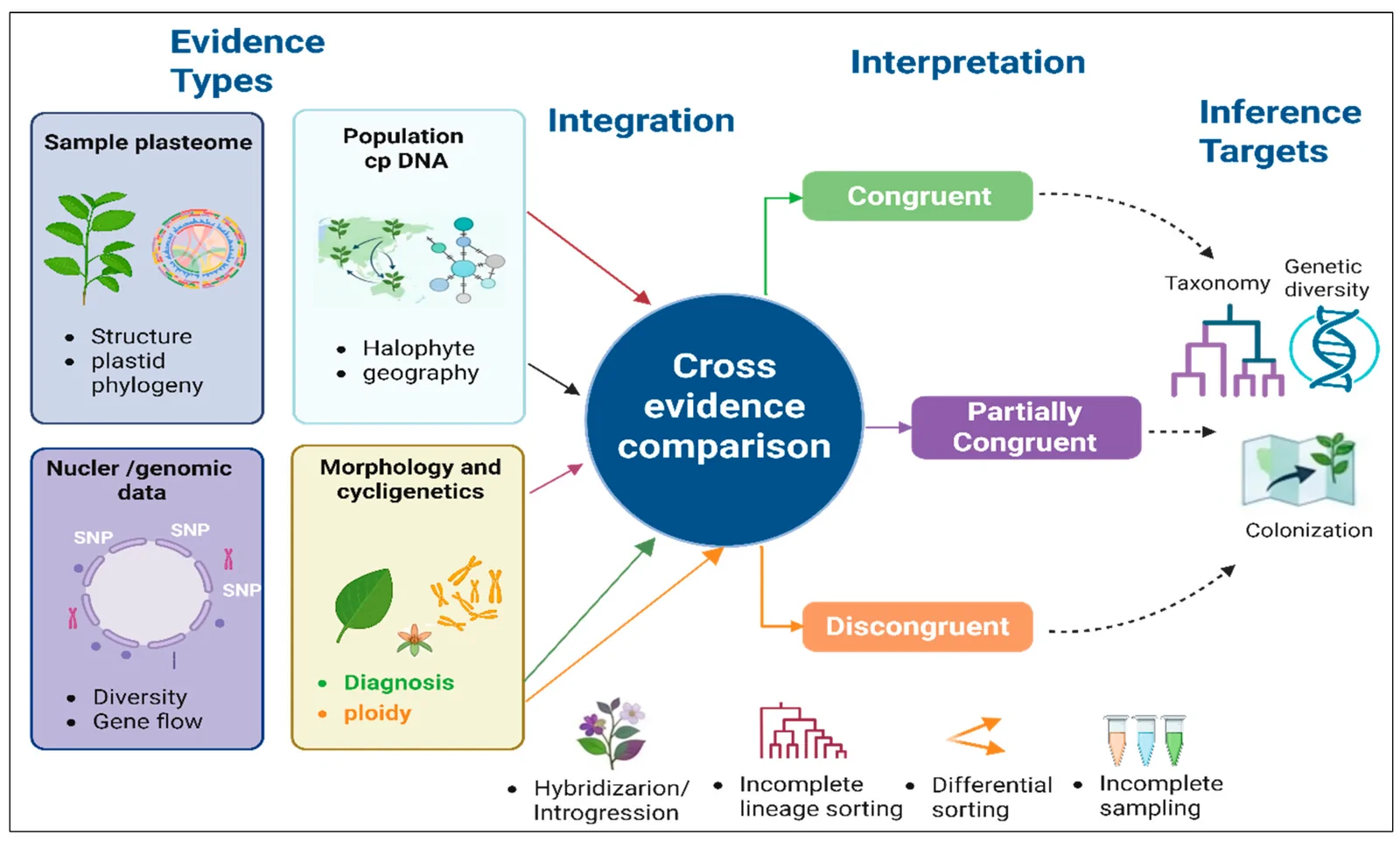
Conceptual framework for evaluating evolutionary evidence in Ulleungdo endemic plants. The schematic illustrates how plastid, nuclear, morphological, and cytogenetic evidence are integrated and classified as congruent, partially congruent, discordant, or unresolved before inference about taxonomy, colonization history, genetic diversity, or evolutionary processes.

This review classifies evidence as congruent when plastid, nuclear, and phenotypic datasets support the same interpretation; partially congruent when datasets support taxon distinctness but differ in ancestry or population history; and discordant when independently inherited datasets support conflicting relationships [39]. This framework separates evidence for taxonomic distinction from evidence for colonization history and prevents a single genomic compartment from being treated as a complete representation of species detailed history [40] (Figure 1).

## 4. Colonization Histories and Genetic-Diversity Outcomes

### 4.1. Probable Single Colonization with Marker-Dependent Diversity Retention

#### 4.1.1. *Prunus takesimensis*

*Prunus takesimensis* provides one of the stronger molecular cases for a predominantly single-origin history among Ulleungdo endemics. Cho et al. combined extensive sampling of *Prunus. takesimensis*, *Prunus sargentii*, and related flowering cherries with multiplexed inter-simple sequence repeat genotyping by sequencing (MIG-seq) single-nucleotide polymorphisms (SNPs) and chloroplast DNA (cpDNA) haplotypes. Nuclear SNP analyses recovered *Prunus sargentii takesimensis* as a genetically differentiated lineage and supported its monophyly relative to the sampled continental material. The combined evidence was most consistent with a probable single colonization from a *Prunus sargentii*-related source on the Korean Peninsula, although the precise geographical source could not be identified [7].

The diversity results are more informative when the genomic compartments are considered separately [41]. Chloroplast estimates indicated lower diversity for some measures in the island lineage, whereas the genome-wide nuclear SNP dataset did not show a significant reduction in genetic diversity relative to *Prunus sargentii* [7]. Nuclear analyses also detected no strong geographical structuring among Ulleungdo populations. These findings do not support a simple expectation that long-distance colonization necessarily leaves a persistent genome-wide diversity deficit. They instead indicate that estimates of diversity retention depend partly on genomic compartment and marker properties [42]. The case is therefore best classified as partially congruent with lineage differentiation and a probable single origin, but marker-dependent regarding the magnitude of retained genetic diversity. The unresolved mainland source remains an important limitation because a single-colonization inference is stronger than the available evidence for identifying the exact ancestral population.

#### 4.1.2. *Phedimus takesimensis*

Population-level analyses of *Phedimus takesimensis* revealed a different form of diversity retention. Seo et al. used nuclear ribosomal internal transcribed spacer (nrDNA ITS) sequences and chloroplast noncoding regions to evaluate *P. takesimensis* together with the putative continental relatives *Phedimus kamtschaticus* and *Phedimus aizoon*. *P. takesimensis* showed cpDNA gene diversity of 0.9346 ± 0.0068 and nucleotide diversity (π) of 0.003290 ± 0.001658, descriptively exceeding the corresponding estimates in the sampled *Phedimus aizoon* and *Phedimus kamtschaticus* populations; however, the authors cautioned that uneven sampling among taxa limits direct comparison [8]. The Ulleungdo populations also showed detectable internal genetic structuring.

The inference of a single insular lineage is stronger than the inference of its precise continental progenitor. Subsequent plastome-scale analysis of East Asian Phedimus recovered both *Phedimus aizoon* and *Phedimus kamtschaticus* as polyphyletic complexes, further demonstrating that progenitor assignment is complicated by unresolved species relationships within the subgenus [43,44]. Consequently, the evidence supports differentiation of *P. takesimensis* and broadly retained diversity, but it does not identify a single mainland source with comparable confidence. This case is therefore congruent: chloroplast and nrDNA evidence support the sampled island lineage, whereas its progenitor relationship remains insufficiently resolved.

### 4.2. Multiple Maternal Origins and Chloroplast Non-Monophyly

*R. takesimensis* departs from a simple single-colonization model. An initial population-level cpDNA analysis using the trnL–F region recovered non-monophyly and inferred at least two independent introductions from geographically differentiated source populations [2]. A subsequent study increased phylogeographic resolution by sampling 315 accessions from 35 populations and sequencing five chloroplast noncoding regions. Of 129 recovered haplotypes, 48 occurred exclusively in *R. takesimensis*. The endemic again failed to form a single chloroplast lineage. Most Ulleungdo accessions were associated with *R. crataegifolius* lineages primarily represented on the Korean Peninsula, whereas rarer accessions showed relationships to lineages sampled from the southern Korean Peninsula, Jeju Island, and Japan. Several Chusan accessions represented an additional independent connection to the Korean Peninsula [19].

High haplotype diversity was concentrated in two parts of Ulleungdo, but no clear island-wide geographical population structure was recovered. The original studies interpreted the repeated chloroplast non-monophyly as consistent with multiple maternal introductions. However, this remains restricted to chloroplast history, and alternative processes such as introgression or retention of ancestral plastid variation cannot be excluded without matched nuclear data [45]. Corresponding population-level nuclear genomic data are required to determine whether repeated maternal introductions also produced multiple nuclear ancestries or whether subsequent gene flow generated a more cohesive nuclear lineage. The current evidence therefore complicates the colonization model without, by itself, invalidating the taxonomic recognition of *R. takesimensis*.

### 4.3. Genetic Drift and Loss of Rare Nuclear Variation

*Acer takesimense* shows nuclear patterns consistent with historical drift and loss of rare variation. Takayama et al. analyzed nine nuclear microsatellite loci in the Ulleungdo endemic and the putative continental progenitor *A. pseudosieboldianum*. Bayesian clustering distinguished the two taxa, while *A. takesimense* showed slightly lower overall genetic diversity and possessed less than half the number of private and rare alleles detected in its continental relative. The authors also recovered weak geographical structure among Ulleungdo populations and interpreted the nuclear pattern as consistent with a substantial episode of genetic drift during colonization and speciation [18].

Loss of rare alleles is potentially more sensitive to demographic contraction than broad measures of heterozygosity or allelic diversity [46]. Nevertheless, contemporary microsatellite variation cannot be interpreted as a direct record of the initial founding population. Mutation, drift, gene flow, and population expansion, along with subsequent demographic change, can alter diversity after colonization [47,48]. The Acer evidence therefore supports historical drift and reduced rare variation, rather than direct measurement of the magnitude of the original founder event.

### 4.4. Cross-Taxon Synthesis

*P. takesimensis* and *P. takesimensis* broadly support probable single-lineage origins accompanied by substantial retention of contemporary genetic diversity [46], although marker resolution and progenitor identification differ between them. *R. takesimensis* instead records multiple chloroplast lineages and repeated maternal colonization [2]. Acer shows substantial nuclear differentiation, along with depletion of rare and private alleles, consistent with historical drift. These outcomes demonstrate that presumed anagenetic speciation on Ulleungdo does not generate a uniform genetic signature. They also differ in evidentiary strength: Prunus integrates genome-wide nuclear SNPs with cpDNA, Phedimus combines chloroplast regions with nrDNA ITS, Rubus remains dominated by chloroplast evidence, and *A. takesimense* is based primarily on nuclear microsatellites [8,18]. Table 1 extends this comparison to *Dystaenia takesimana*, *Campanula takesimana*, and *Acer okamotoanum*, which provide additional question-specific evidence but have more restricted cross-compartment coverage. Cross-taxon comparison must therefore distinguish biological heterogeneity from differences created by marker choice and sampling resolution.

The observed discrepancies likely reflect both biological and methodological heterogeneity. Island genetic patterns can be shaped by drift, mutation, recombination, hybridization, demographic history, and island history [49], while evolutionary inference also depends on genomic compartment, marker choice, and sampling design [50]. Thus, no single genetic signature should be assumed for anagenesis across Ulleungdo: *P. takesimensis* is consistent with probable single colonization [7], *R. takesimensis* retains multiple chloroplast lineages consistent with multiple maternal origins [19], and *H. maxima* shows cytonuclear discordance and introgression [20].

**Table 1:** Comparative evidence for colonization history and genetic-diversity outcomes in principal Ulleungdo endemic lineages.

Taxon	Plastid Evidence	Nuclear Evidence	Key Empirical Findings	Evidence Status	Supported Inference	Ref
*Prunus takesimensis*	Population-level cpDNA haplotypes	Genome-wide MIG-seq SNPs	Monophyletic; differentiated from *P. sargentii*;	Partially congruent	Probable single colonization; exact source unresolved; diversity marker-dependent.	[7]
*Phedimus takesimensis*	Chloroplast noncoding regions	nrDNA ITS	Monophyletic; diversity retained;	Congruent	Single insular lineage supported; progenitor identity remains uncertain.	[8]
*Rubus takesimensis*	Population-level cpDNA	No matched nuclear hypothesis-testing dataset	Chloroplast non-monophyly; 48 endemic haplotypes;	Unresolved	Multiple maternal origins/repeated colonization supported; nuclear history unresolved.	[2,19]
*Acer takesimmense*	No matched plastid hypothesis-testing dataset	Nine nuclear microsatellites	Distinct lineage; reduced rare/private alleles	Unresolved	Historical drift and loss of rare nuclear variation are supported.	[18]
*Dystaenia takesimana*	cpDNA trnL–F	nrDNA ITS + AFLP	Distinct from *D. ibukiensis*; higher AFLP diversity;	Congruent	Consistent with anagenetic divergence and post-colonization diversity accumulation.	[51]
*Campanula takesimana*	Four population-level cpDNA noncoding regions	No matched nuclear hypothesis-testing dataset in focal study	Lower cpDNA diversity; significant structure	Unresolved	Chloroplast evidence supports a restricted source history; nuclear confirmation is lacking.	[52]
*Acer okamotoanum*	No matched plastid hypothesis-testing dataset	Eight nuclear microsatellites	Distinct from *A. mono*; drift	Unresolved	Consistent with a single-origin anagenetic scenario accompanied by historical drift.	[53]

“No matched hypothesis-testing dataset” indicates absence of an independent genomic compartment.

## 5. Morphological Differentiation and Taxonomic Congruence

### 5.1. Multidataset Support for Taxonomic Distinction: A. ulleungense

*A. ulleungense* provides the clearest example in which morphological, cytogenetic, plastid, and nuclear evidence converge on taxonomic distinction. Choi et al. described the Ulleungdo lineage as a separate species from the morphologically similar *A. microdictyon* and *A. ochotense*. The diagnostic characters included broader leaves and larger, whitish perianths. Cytological analysis documented a diploid chromosome complement of 2n = 2x = 16 in *A. ulleungense* [38]. This chromosome count is treated here as supporting cytological characterization rather than as independently diagnostic evidence, because chromosome-number agreement alone does not establish cytogenetic differentiation without demonstrated differences in ploidy, karyotype structure, or chromosome-specific features [36]. This combination of diagnostic morphology, chromosome data, and independently inherited molecular markers provides stronger support for species recognition than plastid phylogenetic placement alone.

Subsequent genomic authentication reinforced this distinction while also revealing a limitation of organellar identification. Lee et al. compared complete plastomes and 45S nuclear ribosomal DNA (nrDNA) among *A. ulleungense*, *A. microdictyon*, *A. ochotense*, and cultivated collections. Lee et al. sequenced plastomes and 45S nrDNA from six collections, comprising representatives of *A. ulleungense*, *A. microdictyon*, and *A. ochotense* and three cultivated collections, and subsequently genotyped 49 collections using ten diagnostic markers. Between *A. ulleungense* and *A. microdictyon*, they reported >170 plastome SNPs and 80 InDels, together with 20 SNPs and one InDel in 45S nrDNA [54]. These sequence differences provide strong diagnostic molecular evidence for authentication but are not, by themselves, sufficient to establish species boundaries. The taxonomic recognition is instead supported by their concordance with independent morphological and molecular evidence.

The cultivated material was more complex. Farm-JB and Farm-SA possessed *A. microdictyon*-type plastomes but showed heterozygous 45S nrDNA sites containing sequence states associated with both *A. microdictyon* and *A. ulleungense*. Lee et al. therefore interpreted these accessions as putative hybrids, an inference further supported by targeted genotyping with seven plastome-derived and three nrDNA-based markers [54]. However, heterozygous nrDNA sites are not independently diagnostic of hybrid ancestry because nrDNA is multicopy and can retain intra-individual polymorphism or undergo concerted evolution [55,56]. Because cloning of nrDNA variants and independent genome-wide nuclear-marker analyses were not reported, these accessions should be regarded as putative rather than confirmed hybrids.

### 5.2. Morphological Distinction but Unresolved Species Delimitation: Viola ulleungdoensis

The status of *V. ulleungdoensis* is less secure because morphological and plastome evidence currently leads to different taxonomic interpretations. The original species description differentiated *V. ulleungdoensis* from *V. selkirkii* using several vegetative characters. *V. ulleungdoensis* lacked adventitious buds, developed larger leaves after flowering, attained a larger vegetative size, and occurred in lower-elevation habitats, whereas *V. selkirkii* was characterized by adventitious buds, smaller leaves that did not enlarge comparably after flowering, and higher-elevation occurrence. The original study also reported molecular differentiation from related Viola taxa [57].

The inference remains limited by sampling. Only one *V. ulleungdoensis* plastome and three *V. selkirkii* individuals were compared, and a plastome phylogeny tests chloroplast relationships rather than genome-wide species boundaries [58,59]. The available evidence therefore raises a synonymy hypothesis but does not constitute a population-level species-delimitation analysis [58]. Resolving taxonomic uncertainty broader sampling of both morphotypes, matched nuclear and plastid data from the same individuals, and quantitative assessment of the diagnostic morphological characters [60]. *V. ulleungdoensis* is consequently classified as taxonomically unresolved rather than demonstrated to be either distinct or synonymous.

### 5.3. Distinctive Morphology with an Unresolved Genomic Basis: Cirsium nipponicum

*C. nipponicum* presents a different problem: conspicuous morphological differentiation has been documented, but its genomic basis has not been established. The Ulleungdo thistle was characterized as having absent or strongly reduced spines relative to the prominently spiny condition typical of many congeners. Plastome analysis placed *C. nipponicum* closer to the sampled *C. arvense* and *C. vulgare* than to the sampled Korean congeners, leading Kim et al. to suggest a northern Eurasian connection [61]. However, limited taxon sampling and reliance on plastid data mean that its geographical origin remains provisional and requires broader taxon sampling with independent nuclear evidence. That biogeographic interpretation should remain provisional. The plastome comparison included only a limited subset of Cirsium diversity, and chloroplast affinity does not independently establish the geographical source of the founding population. Broader nuclear and population-level sampling of plausible continental relatives is required before a northern Eurasian colonization route can be evaluated rigorously [62].

A nuclear reference genome has now been assembled for *C. nipponicum*. The 929.4-Mb assembly contained 31,263 predicted protein-coding genes and provides an important resource for comparative genomic analysis [63]. However, the reference genome does not itself identify the genetic determinants of spine reduction. Gene-family analyses reported expansions and contractions in several functional categories, but no experimental or comparative evidence linked specific variants to the reduced-spine phenotype. The morphological novelty should therefore not be interpreted as a demonstrated genomic adaptation. Functional association will require comparative population genomics, phenotype–genotype analysis, expression studies, or experimental validation.

**Figure 2:**
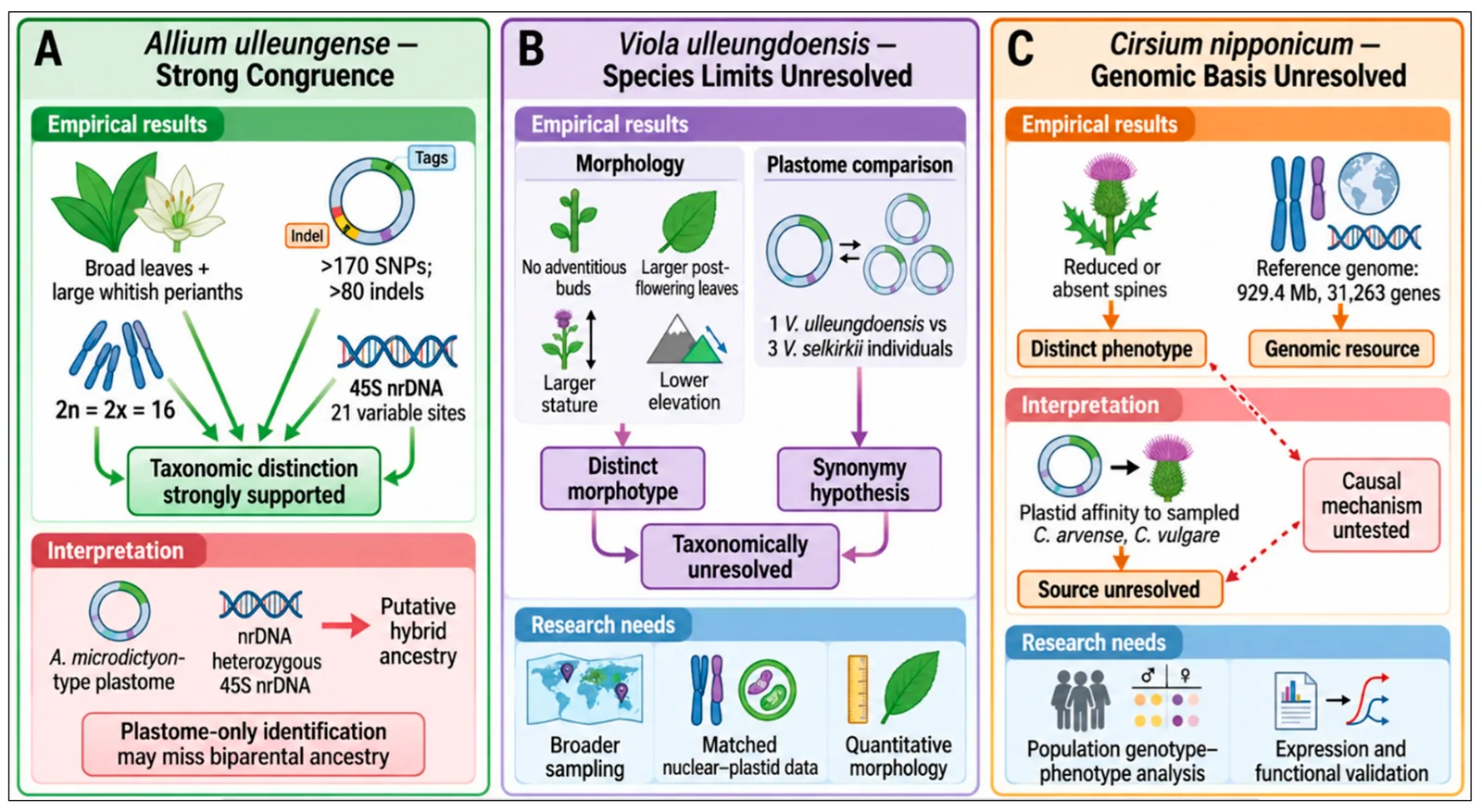
Comparative evidence synthesis for taxonomic and evolutionary inference in selected Ulleungdo endemic plants. (**A**) Independent morphological, cytogenetic, plastid, and nuclear evidence supports the taxonomic distinction of *Allium ulleungense*; (**B**) limited plastome sampling and morphological differentiation leave the species boundary of *Viola ulleungdoensis* unresolved; and (**C**) the reduced-spine phenotype of *Cirsium nipponicum* is established, whereas its source lineage and causal genomic basis remain unresolved.

These taxa represent different levels of morphological–genomic resolution, ranging from strong multidataset support for taxonomic distinction in *A. ulleungense* [54] to unresolved species delimitation in *V. ulleungdoensis* [58] and an unresolved genomic basis of phenotypic differentiation in *C. nipponicum* [61]. Collectively, they show that taxonomic distinctness, phylogenetic placement, and causal genomic mechanisms are separate inferential targets and should not be treated as equivalent conclusions [64,65] (Figure 2).

## 6. Cytonuclear Discordance and Reticulate Evolution

### 6.1. Multicompartment Phylogenomic Discordance in H. maxima

*H. maxima* provides the strongest direct evidence that organellar and nuclear histories can differ in an Ulleungdo endemic lineage. Park and Park generated plastid, mitochondrial, and transcriptomic data and constructed phylogenomic datasets comprising 76 plastid, 37 mitochondrial, and 413 nuclear genes. Concatenation- and coalescent-based analyses recovered conflicting relationships among the three genomic compartments rather than a single concordant species topology [20]. This result is important because the discordance was identified by directly comparing independently inherited genomic datasets rather than by comparing isolated marker studies.

Species-network analyses further supported a history of reticulation within Asian Hepatica. Park and Park inferred both ancient and more recent introgression involving the lineage leading to *H. maxima* [20]. The results therefore weaken a strictly bifurcating interpretation in which the Ulleungdo lineage originated from a single continental progenitor in complete isolation. However, phylogenetic discordance alone should not be attributed exclusively to hybridization. Incomplete lineage sorting can also generate disagreement among gene trees, and organellar datasets contain fewer independently segregating loci than hundreds of nuclear genes [66,67]. The network evidence strengthens the introgression hypothesis, but the relative contributions of introgression and lineage sorting require careful distinction. The proposed adaptive significance of introgressed variation requires similar restraint. The authors suggested that introgression could have supplied genetic variation relevant to adaptation on Ulleungdo, but they did not provide functional or population-genomic evidence directly linking introgressed alleles to adaptive phenotypes [20]. Introgression is therefore supported as an evolutionary process, whereas its adaptive benefit remains a hypothesis.

### 6.2. Unresolved Ancestry and Possible Hybrid Origin of Fagus multinervis

*F. multinervis* shows a different form of discordance. Morphologically, the Ulleungdo beech can be distinguished from the closely related *F. engleriana* by characters such as vertically elongated rhombic lenticels. Molecular evidence also supports recognizing *F. multinervis* as a distinct insular lineage, but its ancestry remains unresolved [60].

Population-level chloroplast sampling revealed an especially restricted organellar pattern. Sequencing of the chloroplast psbA–trnH region from 144 individuals detected no haplotype variation within *F. multinervis*. This absence of chloroplast haplotype diversity, in contrast to previously reported high allozyme diversity, indicates markedly different diversity patterns between organellar and nuclear-coded markers [68,69]. Several demographic histories could generate such a pattern, and the chloroplast data did not discriminate uniquely among them.

More direct phylogenetic conflict emerged when four plastid regions were compared with the nuclear LEAFY second intron. *F. multinervis* was recovered as monophyletic and closely related to *F. engleriana* and *F. japonica*, but plastid and nuclear relationships were incongruent. Oh et al. interpreted this discordance as compatible with a possible hybrid origin [70]. The available evidence is insufficient to demonstrate hybrid speciation because the nuclear component was represented by a single locus, and alternative explanations, including incomplete lineage sorting, remain plausible [71]. Plastome-scale analysis subsequently supported the taxonomic distinction of *F. multinervis* from *F. engleriana* and *F. japonica* but did not resolve its sister species or geographical source. Nuclear phylogenomics based on 28 single- or low-copy loci likewise recognized *F. multinervis* as an independent evolutionary unit [72,73]. Thus, taxonomic distinctness is better supported than any specific hypothesis regarding progenitor identity or hybrid origin.

### 6.3. Recurrent Hybrid Formation in Viola woosanensis

*V. woosanensis* provides a more directly resolved example of reticulate evolution. Morphology had suggested a hybrid origin between *V. ulleungdoensis* and *V. chaerophylloides*. Gil and Kim tested this hypothesis using 80 accessions, nuclear ribosomal DNA internal transcribed spacer (nrDNA ITS) sequences, and four chloroplast noncoding regions. Chloroplast haplotypes were interpreted as supporting *V. ulleungdoensis* as the maternal contributor, assuming maternal plastid inheritance, whereas additive nrDNA ITS polymorphisms supported *V. chaerophylloides* as the other parental lineage [74,75].

Most sampled hybrids retained both parental ribotypes and were interpreted as predominantly early-generation, mainly F1, hybrids. The study detected no clear evidence of backcrossing or introgression into the parental species and associated this pattern with low hybrid fertility. Multiple independent hybridization events were inferred, but the direction of chloroplast inheritance was consistently from *V. ulleungdoensis*, supporting recurrent unidirectional formation [74]. Unlike the provisional hybrid hypothesis for *F. multinervis*, the *V. woosanensis* interpretation is supported by congruent morphological evidence and complementary biparental and chloroplast markers [76,77].

### 6.4. Inferential Consequences

Gene-tree discordance refers to conflicting phylogenetic relationships among loci or genomic compartments and may arise from incomplete lineage sorting, hybridization/introgression, recombination, or analytical factors such as orthology and gene-tree estimation errors [78]. Species non-monophyly, by contrast, describes the failure of sampled members of a species to form an exclusive clade within a particular gene tree and may result from retained ancestral polymorphism, introgression, and taxonomic limitations [79]. In *R. takesimensis*, the observed pattern is specifically chloroplast non-monophyly, which was interpreted as evidence of multiple maternal origins; however, matched nuclear data are required to distinguish this scenario from introgression or retention of ancestral plastid variation [19]. Distinguishing among these processes requires matched nuclear and organellar sampling [80,81], preferably at population scale, together with phylogenetic-network or explicit gene-flow analyses [82]. For Ulleungdo endemics, reticulate evolution is therefore not a peripheral complication but a process that must be tested before plastid relationships are interpreted as direct evidence of anagenetic species history (Table 2). Several evolutionary histories therefore remain unresolved because progenitor sampling, matched nuclear–plastid data, population-level coverage, or ecological and reproductive evidence are insufficient to distinguish among alternative hypotheses. These cases are treated as explicit knowledge gaps rather than definitive evolutionary conclusions.

## 7. Conservation Interpretation and Research Priorities

### 7.1. Defensible Applications of Plastome Evidence

Plastome data have several defensible conservation applications when their lineage-specific inheritance and organellar genealogy are appropriately considered. Population-level chloroplast sampling can identify chlorotypes, reconstruct chloroplast-lineage relationships, and test seed-mediated dispersal or colonization hypotheses where plastid inheritance is established [83,84]. In *R. takesimensis*, geographically structured chloroplasts were interpreted as consistent with multiple maternal contributions to the Ulleungdo lineage, demonstrating the value of population-level plastid data for reconstructing colonization history [19]. Plastomes can also provide diagnostic sequence variation for germplasm authentication and marker development [85]. In *A. ulleungense*, combined plastome and 45S nuclear ribosomal DNA (nrDNA) analyses distinguished authenticated taxa and identified cultivated accessions with putative hybrid ancestry that plastome data alone could not resolve [54]. Thus, plastomes are useful for identifying chloroplast lineages and flagging accessions that require nuclear-genomic investigation.

### 7.2. Limits for Conservation Decisions

These applications should not be extended to parameters that depend primarily on biparentally inherited, genome-wide variation. Plastome diversity alone does not provide direct estimates of nuclear heterozygosity, genomic inbreeding, effective population size, nuclear admixture, or adaptive genetic variation. Nor can plastid differentiation by itself establish conservation-management units when nuclear connectivity and genome-wide structure remain unknown. Population genomic studies illustrate the additional information required: whole-genome single-nucleotide polymorphisms and structural variants have been used jointly to estimate inbreeding, reconstruct effective population-size histories, resolve population structure, and delineate conservation units in threatened plants [86,87]. For Ulleungdo endemics, the discordant plastid and nuclear histories recovered in *H. maxima* further show why organellar relationships should not be translated directly into conservation units [20]. Accordingly, this review does not designate management units or prescribe taxon-specific conservation interventions. Such decisions require robust evidence on genome-wide population structure, connectivity, demographic history, ecological differentiation, and threats; until these data are available, the conservation implications remain provisional [88].

### 7.3. Prioritized Research Tests

Future research should address current evidence gaps through complementary approaches selected according to the unresolved question and available evidence for each taxon. These include population-level plastid sampling to characterize chlorotype diversity and geographic structure, matched nuclear–plastid datasets to test cytonuclear concordance and introgression, standardized morphometric analyses of genomically characterized individuals, and broader continental sampling to evaluate progenitor relationships. Common-garden experiments, phylogenetic-network or genomic-cline analyses, and functional validation should be applied where phenotypic plasticity, hybridization, or adaptive mechanisms are specifically hypothesized. Environmental evidence should also be integrated with genomic and phenotypic data. Analyses of climatic and edaphic variation, ecological niche differentiation, and genotype–environment associations can help evaluate whether environmental heterogeneity contributes to lineage divergence beyond demographic and geographic effects [1,89]. Where feasible, common-garden or reciprocal-transplant experiments can further distinguish genetically based differentiation from environmentally induced phenotypic variation.

**Figure 3:**
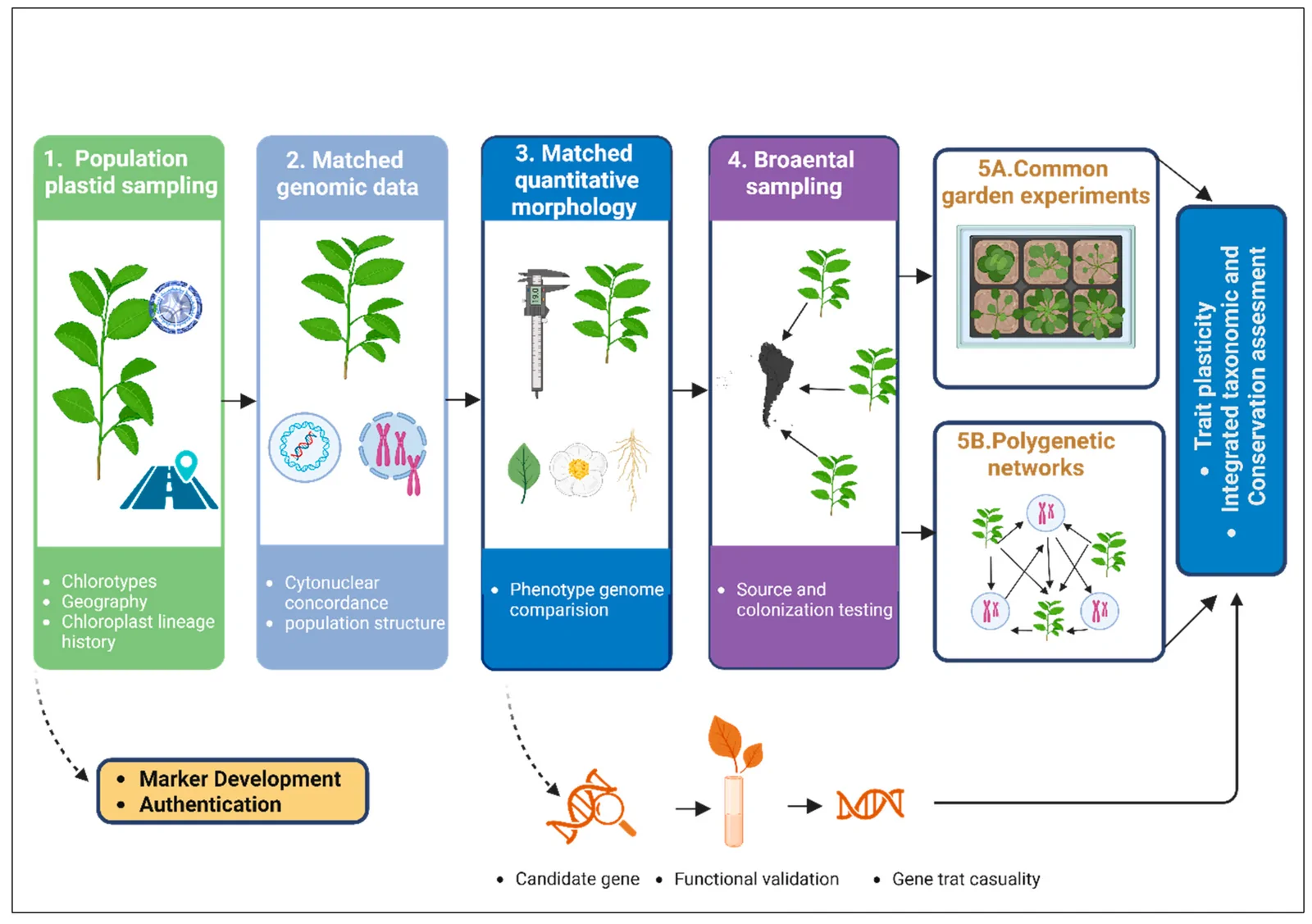
Proposed research priorities for strengthening evolutionary, taxonomic, and conservation inference in Ulleungdo endemic plants. The schematic summarizes prospective research steps, including population-level plastid sampling, matched nuclear–plastid datasets, quantitative morphology, broader source-population sampling, common-garden experiments, phylogenetic-network analyses, and functional validation. These elements represent recommended future tests rather than empirical evidence generated in this review.

Taxon-specific priorities differ according to the current evidence—for instance, *P. takesimensis* requires broader mainland-source sampling, whereas *P. takesimensis* and *Acer takesimense* require expanded population-genomic sampling to resolve progenitor relationships and determine whether marker-based signals of structure or drift extend across the nuclear genome; whole-genome resequencing has proven effective for resolving population structure, demographic history, and conservation-relevant genomic variation in endangered plants [90,91]. The R takesimensis requires matched nuclear sampling across chloroplast-diverse populations to determine whether maternal-lineage structure corresponds to genome-wide differentiation. Population-level nuclear–plastid analyses are likewise priorities for *V. ulleungdoensis* and *C. nipponicum*, whereas *H. maxima* and *F. multinervis* require population-genomic analyses of connectivity, introgression, and cytonuclear structure. Such genome-wide approaches can reveal geographically structured lineages and gene flow that are not resolved by single-marker datasets [92]. *A. ulleungense* additionally requires genetic authentication of wild and cultivated material where hybrid ancestry is suspected. Accordingly, candidate management units should remain provisional until genome-wide population structure, connectivity, and demographic differentiation are demonstrated; whole-genome studies in threatened plants show that such information can substantially refine the delimitation of conservation units and management priorities [93,94] (Figure 3).

## 8. Conclusions

Evidence across Ulleungdo endemics does not support a uniform genetic consequence of island colonization or presumed anagenetic speciation. The strength of evidence for anagenesis varies substantially among lineages and remains provisional where progenitor identity, alternative colonization scenarios, or cross-compartment evolutionary histories have not been adequately resolved. Probable single-origin histories occur in taxa such as *P. takesimensis*, whereas *R. takesimensis* retains evidence of multiple maternal origins, and *Acer takesimense* shows nuclear signatures consistent with historical drift and loss of rare alleles. Morphological and genomic evidence are strongly congruent in *A. ulleungense*, whereas other taxa retain unresolved taxonomic relationships or genuine phylogenetic discordance. Multicompartment analyses of *H. maxima* further demonstrate that introgression can decouple plastid and nuclear histories. Plastomes therefore remain valuable records of chloroplast lineage history, but robust inference of species boundaries, morphological evolution, and conservation significance requires integration with nuclear, phenotypic, reproductive, and population-level evidence. Plastomes are informative for chloroplast lineage history, but robust evolutionary and conservation inference requires integration with nuclear, phenotypic, reproductive, population-level, and environmental evidence.

## Figures and Tables

**Table 2:** Cross-compartment congruence, discordance, and unresolved evolutionary inference in selected Ulleungdo plant lineages.

Taxon	Evidence Base	Nuclear Evidence	Morphology Cytogenetics	Evidence Status	Key Empirical Findings	Ref
*Allium ulleungense*	Plastid markers + plastome	Nuclear markers + 45S nrDNA	Diagnostic morphology; 2n = 2x = 16	Congruent (taxonomy)	Molecular and phenotypic differentiation; putative cultivated hybrids	[38,54]
*Viola ulleungdoensis*	Limited comparative plastomes	No matched population-level nuclear dataset	Diagnostic vegetative morphology	Unresolved		[57]
*Cirsium nipponicum*	Comparative plastome	929.4-Mb reference genome (resource)	Reduced/absent spines	Unresolved	Morphological differentiation; Ulleungdo *V. selkirkii* plastome groups with *V. ulleungdoensis*	[61]
*Hepatica maxima*	76 plastid + 37 mitochondrial genes	413 nuclear genes	—	Discordant	Organellar and nuclear relationships conflict	[20]
*Fagus multinervis*	Population cpDNA + plastid loci/plastome	LEAFY intron + 28 nuclear single/low-copy loci	Diagnostic lenticel morphology	Discordant (ancestry)	No psbA–trnH variation in 144 individuals; plastid–nuclear relationships conflict	[68,70]
*Viola woosanensis*	Four cpDNA noncoding regions	nrDNA ITS + cloned ribotypes	Morphology consistent with hybrid origin	Congruent (hybrid origin)	*V. ulleungdoensis* maternal; *V. chaerophylloides* paternal	[74]

Genomic resource denotes sequence information that does not independently test the focal evolutionary hypothesis. Unresolved denotes insufficient or missing evidence.

## Data Availability

No new data were created or analyzed in this study. Data sharing is not applicable.
